# Methicillin-resistant *Staphylococcus aureus* risk profiling: who are we missing?

**DOI:** 10.1186/2047-2994-2-17

**Published:** 2013-05-30

**Authors:** Janet Pasricha, Stephan Harbarth, Thibaud Koessler, Veronique Camus, Jacques Schrenzel, Gilles Cohen, Didier Pittet, Arnaud Perrier, Anne Iten

**Affiliations:** 1 Infection Control Program, University of Geneva Hospitals and Faculty of Medicine, 4 Rue Gabrielle Perret-Gentil, Geneva 1211, Switzerland; 2 Department of General Internal Medicine, University of Geneva Hospitals and Faculty of Medicine, Geneva, Switzerland; 3 Central Laboratory of Bacteriology, University of Geneva Hospitals and Faculty of Medicine, Geneva, Switzerland; 4 Direction of Medico-Economic Analysis, University of Geneva Hospitals and Faculty of Medicine, Geneva, Switzerland; 5 Current affiliation: The Jenner Institute, Oxford University, Oxford, UK

**Keywords:** Carrier state, Epidemiology, MRSA, Prevalence, Probability, Predictive value of tests, Staphylococcal infection, Switzerland

## Abstract

**Background:**

Targeted screening of patients at high risk for methicillin-resistant *Staphylococcus aureus* (MRSA) carriage is an important component of MRSA control programs, which rely on prediction tools to identify those high-risk patients. Most previous risk studies reported a substantial rate of patients who are eligible for screening, but failed to be enrolled. The characteristics of these missed patients are seldom described. We aimed to determine the rate and characteristics of patients who were missed by a MRSA screening programme at our institution to see how the failure to include these patients might impact the accuracy of clinical prediction tools.

**Findings:**

From March-June 2010 all patients admitted to 13 internal medicine wards at the University of Geneva Hospital (HUG) were prospectively screened for MRSA carriage. Of 1968 patients admitted to the ward, 267 patients (13.6%) failed to undergo appropriate MRSA screening. Forty-one (2.4%) screened patients were MRSA carriers at admission. On multivariate regression, patients who were missed by screening were more likely to be aged < 50 years (OR 2.4 [1.4-3.9]), transferred to internal medicine from another ward in the hospital (OR 2.8 [1.1-7.1]), and have a history of malignancy (OR 3.2[2.1-5.1]). There was no significant difference in the rate of previous MRSA carriage between screened and unscreened patients.

**Conclusions:**

Our findings highlight the potential bias that “missed” patients may introduce into MRSA risk scores. Reporting on the proportions and characteristics of missed patients is essential for accurate interpretation of MRSA prediction tools.

## Findings

### Introduction

Prevention and control of MRSA cross infection is among the most important challenges of infection control. Surveillance of all patients for MRSA carriage on admission to hospital allows those patients colonised with MRSA to be isolated and contact precautions undertaken, with the aim of minimising spread to other patients. As patients with MRSA evident on routine clinical specimens represents a small fraction of the burden of MRSA, surveillance is needed to identify the reservoir of colonised but not infected patients [1,2]. However, universal surveillance utilises significant healthcare resources, and its effectiveness is debatable [3-5]. Despite this, screening is increasingly utilised in hospital MRSA control programs, and is still legislated in the United Kingdom and some states of the USA [6,7]. To mitigate costs without sacrificing the effectiveness of surveillance, many MRSA screening programs rely on clinical prediction tools to target patients at high risk of MRSA carriage [5]. Several epidemiological studies form the basis of these tools in which the major risk factors for MRSA carriage have been identified, including: a history of MRSA colonization, admission to intensive care, hospitalization in the previous 12 months, extensive contact with health care, previous receipt of antibiotic therapy and skin or soft tissue infection at admission [8-12]. However, these studies report 5-83% of patients who were eligible for study, but not screened. The characteristics of these missed patients are seldom described [11]. We examined the characteristics of patients who were missed during a MRSA surveillance study at our institution to ascertain whether their exclusion might introduce bias and affect the accuracy of clinical prediction tools and risk profiling. Specifically, we hypothesised that an important proportion of patients would be missed by our MRSA screening programme, and that these patients would differ from those patients who were not missed.

### Setting and methods

The University of Geneva Hospitals (HUG) are a 2200-bed tertiary hospital network providing in- and outpatient care to the Canton of Geneva. From March to June 2010 a universal MRSA surveillance program was undertaken to prospectively screen all patients consecutively admitted to 13 internal medicine wards. The primary aim of this study was to determine the rate of MRSA carriage amongst patients admitted to internal medicine. Secondary aims included: to formulate a clinical prediction tool that would accurately predict those patients at high risk of MRSA carriage on admission to internal medicine, and to: determine the effectiveness of our programme to capture all patients for screening. Over the study period, all patient admissions to internal medicine were recorded and basic demographic and clinical data was collected. Further clinical data were obtained by retrospectively accessing electronic medical records. All patients >18 years of age were eligible for screening and were screened for MRSA by pooled nose and groin swabs. Trained ward nurses conducted the screening seven days a week. Pooled samples were streaked onto MRSAid agar (bioMérieux, Lyon, France) and then inoculated into a colistin-salt (CS) broth. When no MRSA was detected on chromogenic agar at day 1, a second MRSAid plate was inoculated using the overnight enrichment in the CS broth. Suspect colonies were confirmed by a duplex polymerase chain reaction to assess the presence of the *mecA* gene [13].

The proportion of patients who were eligible for, but did not have MRSA screening was determined. Wilcoxon rank sum tests and chi^2^-tests were used to assess differences between screened and unscreened groups. Factors potentially associated with failure to screen were first evaluated using univariate logistic regression. Variables with a *P* value <0.2 were retained. Multivariate models were then developed and variables were eliminated in a stepwise fashion using likelihood ratio tests to compare each model to the previous one (STATA 11.2; StataCorp, College Station, Texas, USA).

## Results

Of 1968 patients admitted to internal medicine, 1740 (88.4%) underwent admission screening within 48 hours of admission. 228 (11.6%) admitted patients were not screened and 39 (2.0%) patients underwent screening but not within 48 hours of admission. Therefore, 267 patients (13.6%) failed to undergo appropriate MRSA screening. Forty-one (2.4%) screened patients were MRSA carriers at admission. Patients who were missed during MRSA screening were younger (57.1 years vs 61.6 years; *P* < 0.0001) and a greater percentage had been transferred to internal medicine from another hospital ward (7.0% vs 2.7%; *P* < 0.0001). The proportions of patients identified as previous MRSA carriers was not significantly different between the screened and unscreened groups (9.6% vs 13.2%, respectively, *P* = 0.308). There was no significant difference in the proportion of patients missed by screening on weekends as compared to weekdays. The results of uni- and multivariate regression analysis of factors potentially associated with being missed for MRSA screening are shown in the Table 1. On multivariate regression, patients who were missed by screening were more likely to be aged < 50 years, admitted to internal medicine from another hospital, and have a history of malignancy.

**Table 1:** Results of uni- and multi-variate regression analyses of factors associated with failure to have an admission MRSA swab performed^1^

	**Proportions (n)**		**Univariate regression**		**Multivariate regression**	
**Variable**	**Swabs missed (n = 267)**	**Swabs done (n = 1740)**	**OR [95%CI]**	***P *value**	**Adjusted OR [95%CI]**	***P *value**
Male sex	55.9 (148)	58.6 (997)	0.9 [0.7-1.1]	0.402		
Age < 50 years old	30.3 (81)	19.9 (339)	1.7 [1.3-2.3]	<0.001	2.4 [1.4-3.9]	<0.001
Admitted from ICU	4.5(12)	7.6(129)	0.6 [0.3-1.1]	0.074		
Admitted from another ward	7.1(19)	2.7(45)	2.8 [1.6-4.9]	<0.001	2.8 [1.1-7.1]	0.028
Admitted from home	88.3(235)	89.7(1526)	0.9 [0.6-1.3]	0.484		
Uncomplicated diabetes mellitus	10.4(12	18.7(126)	0.5 [0.3-0.9]	0.034		
Acute renal failure	10.4(12	21.8(147)	0.4 [0.2-0.8]	0.006		
Chronic renal failure	6.1(7)	13.8(93)	0.4 [0.2-0.9]	0.026		
End stage renal failure	4.3(5)	2.4(16)	1.9 [0.7-5.2]	0.231		
Complicated diabetes mellitus	5.2(6)	9.8(66)	0.5 [0.2-1.2]	0.122		
Peripheral vascular disease	2.6(3)	4.3(29)	0.6 [0.2-2.0]	0.400		
Chronic obstructive pulmonary disease	9.6(11)	14.4(97)	0.6 [0.3-1.2]	0.167		
Dementia	0.9(1)	1.5(10)	0.6 [0.07-4.6]	0.608		
Stroke	0	0.9(6)	Omitted			
Cerebral haemorrhage	1.0(1)	0.4(3)	2.0 [0.2-19.0]	0.561		
Congestive cardiac failure	11.3(13)	24.8(167)	0.4 [0.2-0.7]	0.002		
Ischaemic heart disease	10.4(12)	11.3(76)	0.9 [0.5-1.8]	0.791		
Haematological malignancy	10.4(12)	5.8(39)	1.9 [1.0-3.7]	0.065		
Carotid artery stenosis	0	1.2(8)	Omitted			
Parkinsons disease	0.9(1)	0.7(5)	1.2 [0.1-10.2]	0.884		
Connective tissue disease	0.9(1)	2.2(15)	0.4 [0.05-2.9]	0.358		
Liver failure	1.0(1)	1.8(12)	0.5 [0.06-3.8]	0.488		
Respiratory failure	0	1.0(7)	Omitted			
Solid organ cancer	35.7(41)	15.4(104)	3.0 [1.9-4.7]	<0.001	3.2[2.1-5.1]	<0.001
Metastatic cancer	12.6(13)	7.3(50)	1.8 [0.3-3.9]	0.866	2.6 [1.4-4.8]	0.004
Peptic ulcer disease	1.9(2)	2.5(17)	1.1 [0.3-3.9]	0.866		
Infection	1.9(2)	0.7(5)	2.4 [0.5-12.5]	0.301		
Day of the week						
Monday	8.90(35)	91.10(358)	0.8[0.4-1.4]	0.355		
Tuesday	16.02(62)	83.98(325)	1.5[0.9-2.5]	0.140		
Wednesday	15.93(54)	84.07(285)	1.5[0.9-2.5]	0.155		
Thursday	16.08(46)	83.92(240)	1.5[0.9-2.6]	0.153		
Friday	15.49(35)	84.51(191)	1.4[0.8-2.6]	0.226		
Saturday	8.61(13)	91.39(138)	0.7[0.4-1.5]	0.408		
Sunday	11.35(21)	88.65(164)	0.8[0.5-1.3]	0.365		

^1.^ Denominators used to calculate proportions were adjusted for the number of patients in whom data were available. *OR* odds ratio, *CI* confidence interval.

## Discussion

Screening patients for MRSA carriage on admission to hospital is an increasingly important component of hospital MRSA control programs. Many programs rely on prediction tools so that patients at high risk of MRSA carriage may be targeted for selective screening rather than to utilise universal screening which is costly and resource intensive. Ideally, prediction tools are formulated using local epidemiological data from (universal) surveillance studies. However, many of these studies report a substantial rate of patients who are eligible for screening, but fail to be enrolled by the surveillance programme. The characteristics of these patients are seldom described.

In this study, 13.6% of patients failed to have admission MRSA screening swabs performed. This rate of “missed” screening opportunities is comparable to that found in other MRSA risk profiling studies [8-11,14]. Patients who were not screened differed from those who were in several ways. Firstly, younger patients (<50 years) were more likely to be missed during MRSA screening. A possible explanation for this is that nurses perceived younger patients to be at low risk for MRSA carriage and were thus less inclined to pursue screening. Although older age is frequently identified as a risk factor for MRSA carriage [8,10,12], it is possible that the tendency to miss younger patients from screening may contribute to this finding and inflate effect estimates. Transfer to internal medicine from another hospital department (intra-hospital transfer) was also a risk factor for being missed during screening. Intra-hospital transfer has been previously identified as a risk factor for MRSA admission carriage [10]; missing this group of patients could result in an underestimation of the true MRSA carriage rate and a failure to recognise intra-hospital transfer as an important risk factor for MRSA carriage. Patients with malignancy were more likely to be missed during screening in our study. This was due to logistic difficulties (e.g. frequent readmissions for chemotherapy; ultra-short hospitalizations) within our hospital oncology ward that impeded their regular participation in screening, and was therefore a problem specific to our institution.

To our knowledge, the study by Furano et al. is the only one to report detailed characteristics of patients missed by MRSA screening [11]. In this study, 83.7% of eligible patients were not enrolled in screening. Un-enrolled patients were older, less likely to have had a hospital admission in the previous year, and had a higher in-hospital mortality than those patients who were enrolled [11]. The present study will help to further elucidate the importance and magnitude of misclassification bias in MRSA risk profiling studies.

Our study has several limitations. Firstly, it is likely that the effectiveness of hospital surveillance programmes to enrol patients on admission may be heavily influenced by institutional and local factors. Therefore, the generalizability of our findings may be limited. Secondly, some of our data was collected retrospectively from medical records and is therefore subject to the inaccuracies inherent to data collected in this way.

Nevertheless, we believe that our findings highlight some of the potential misclassification biases that may occur in MRSA risk profiling studies due to patients missed from screening. This could have important implications for the accuracy of MRSA risk scores developed to target MRSA screening. Clear reporting on patient recruitment and the proportions and characteristics of those patients missed is essential for accurate interpretation of clinical prediction tools identifying patients at high risk for carriage of antibiotic-resistant bacteria.

## Competing interests

S. H. has received a peer-reviewed MRSA research grant funded by Pfizer, and is a member of the speakers’ bureau of bioMérieux, and a member of the advisory boards of Destiny Pharma, bioMérieux, and DaVolterra. JS is a consultant for bioMérieux, Biocartis, and Spinomix, and has received research grants and conference support from Abbott, Becton-Dickinson and Bruker.

## Authors’ contributions

AI, TK, AP and DP designed the study, supervised the data collection and contributed to the data analysis. AP and DP provided financial support. JS supervised the laboratory work. VC and GC led data collection and validation. JP performed the data analysis and prepared the manuscript. SH and AI supervised and led the data analysis and manuscript preparation. All authors contributed and approved the final manuscript.
